# Commentary: Rethinking the Development of “Nonbasic” Emotions: A Critical Review of Existing Theories

**DOI:** 10.3389/fpsyg.2015.01967

**Published:** 2016-01-11

**Authors:** Peter Lewinski

**Affiliations:** ^1^Department of Communication, University of AmsterdamAmsterdam, Netherlands; ^2^Faculty of Economics, Université de NeuchâtelNeuchâtel, Switzerland

**Keywords:** basic emotions, non-basic emotions, infants, development of emotions, commentary

Draghi-Lorenz et al. (2001), in their well-cited review article (104 times in Google Scholar and 37 times in Web of Science, as of 07 December 2015), outlined current theories of emotional development in infants. They summarized the arguments of a few well-known researchers in the field, describing both the theoretical approaches and the rationale behind those approaches. In the end, the authors argued against the mainstream chronology describing the emergence of so-called “nonbasic” emotions. According to their critical review, infants younger than 2 years old may have the capacity to experience these emotions.

Draghi-Lorenz et al. first delineated those emotions widely considered “basic,” namely interest, disgust, joy, distress, anger, sadness, surprise, and fear. The nonbasic emotions are shame, embarrassment, coyness, shyness, guilt, jealousy, pride, contempt and so on. Then the researchers further explored the literature surrounding some of the nonbasic emotions, finding a surprising number of studies showing that infants as young as 2 months might be found to express these emotions (Guillaume, 1926; Buhler, 1930; Piaget, 1932; Hoffmann, 1984; Masciuch, 1988; Reissland, 1990; Trevarthen, 1992; Reddy, 2000). For example, Reddy (2000) found that 2–3 month-old infants showed “coy” smiling, which occurs simultaneously with gaze/head aversion and curving arm movement, and had been reported before only in 2-year-old toddlers earliest.

Next, the review presented two contrasting emotional development theorists from two theoretical polarities: the Lewis (Lewis, 1987, 1993) and the Trevarthen (Trevarthen, 1979, 1984) theories. Fundamentally, Lewis's framework may best be illustrated in the context of two basic and interconnected proposals: “The young infant is incapable of ‘nonbasic’ emotions because these depend on specific higher representational skill and this is so because until these skills emerge the infant cannot experience his/her own emotions nor those of others” (Draghi-Lorenz et al., 2001, p. 273).

The key difference between Lewis's and Trevarthen's reasoning lies in Trevarthen's belief that both nonbasic and basic emotions are independent from higher representational skills, which are developed only later in life. Furthermore, Trevarthen holds a nativist point of view on the matter of *when* interpersonal awareness is present (from birth, if not before). Draghi-Lorenz et al. (2001) argued specifically against those two concepts in the conclusion of their review. Instead, they suggested a theoretical construct that synthesizes emotion and representational skills and adopts a different perspective on interpersonal awareness. Conceivably, this approach could explain a great deal about the emergence of nonbasic emotions and account for the large discrepancies between theorists of early emotional development. The authors noted that most psychologists in the field believe that nonbasic emotions emerge when at the age of two. These psychologists assume that a conceptual representation of the self is necessary for the expression of such emotions as pride, guilt, jealousy, etc. and that the capacity for interpersonal awareness develops no sooner than 2 years after birth.

Ultimately, Draghi-Lorenz et al. developed a theoretical frame of reference for early nonbasic emotions to assess the possibility of more complex emotions in infants younger than 2 years, while also giving a nod to theoretical and practical consequences. Importantly, the authors claimed, “interpersonal awareness should be understood as a continuous process rather than as an achievement at some point in time” (p. 296). Therefore, infants' acquisition of the capacity to express increasingly complex emotions should not be seen as a one-time accomplishment but rather as an on-going process starting early in life (possible before birth), finally ending late in adulthood (or possibly never). The authors agreed there is somewhat limited evidence supporting the possibility of early nonbasic emotions, but they elaborated on some other important points stemming from their critical review. They pointed out that it is feasible that infants are aware of others from a quite early age (by showing interest in human features such as face, human voice and movement) (Walker-Andrews, 1997) and that an infant also may have awareness of itself (e.g., Butterworth, 1989, 1995). In the end, Draghi-Lorenz et al. appropriately and importantly noted that paradigm change in the perception of how and when complex (i.e., nonbasic) emotions emerge highlights infants as agents with significantly more competency who are active participants in social interactions.

The shortcomings of Draghi-Lorenz et al.'s review must be acknowledged. Most glaringly, the research presented in their article supporting the early emergence of nonbasic emotions is outdated. Few papers cited are from the Twenty-first century. Many papers represent research from the first half of the Twentieth century, with the earliest citation dating to 1892. In addition, the methodologies for acquiring and analyzing data have evolved dramatically since the middle of the last century. That is, future research in development of infant's emotions could benefit from emotion coding software (e.g., Lewinski et al., 2014a), which can objectively code (based on objective datasets such as Olszanowski et al., 2015) large numbers of facial videos outperforming even human coders under certain circumstances (Lewinski, 2015a). Such software can help in saving substantial amount of coding time (Lewinski et al., 2014b; Lewinski, 2015b). Nonetheless, research after 2001 supports Draghi-Lorenz et al. conclusions. For example: (i) Colonnesi et al. (2013) replicated and extended Reddy (2000) findings to positive shyness; (ii) Hart and Carrington (2002) and Hart et al. (2004) provided new evidence on 6-month-old infants being jealous of mother's attention; (iii) Draghi-Lorenz et al. (2005) found that 2-to-4-month-old infants “can be perceived as shy, coy, bashful or embarrassed” (p. 63).

In conclusion, Draghi-Lorenz et al. were innovative in their review of and challenge to the status quo of procedures and knowledge in the realm of early childhood emotional development. Following the authors' line of reasoning, one may concur that the nonbasic emotions might be present in infants much younger than 2 years old. Nonetheless, without a doubt, more data could be collected that would support the opposite point of view. If a choice must be made, perhaps it is better to consider the infant as “competent neonate” rather than needy and “not-that-sophisticated” beings.

## Funding

The research leading to these results has received funding from the People Programme (Marie Curie Actions) of the European Union's Seventh Framework Programme FP7/2007-2013/ under REA grant agreement 290255.

### Conflict of interest statement

The author declares that the research was conducted in the absence of any commercial or financial relationships that could be construed as a potential conflict of interest.

## References

[B1] Buhler C. (1930). The First Year of Life. New York, NY: John Day.

[B2] Butterworth G. (1989). Events and encounters in infant perception, in Infant Development, eds Slater A. Bremner G. (Hove: Erlbaum), 73–83.

[B3] Butterworth G. (1995). An ecological perspective on the origins of self, in The Body and the Self, eds Bermudez J. L. Marcel A. Eilan N. (Cambridge, MA: MIT Press), 87–105.

[B4] Colonnesi C. Bögels S. M. de Vente W. Majdandžić M. (2013). What coy smiles say about positive shyness in early infancy. Infancy 18, 202–220. 10.1111/j.1532-7078.2012.00117.x

[B5] Draghi-Lorenz R. Reddy V. Morris P. (2005). Young infants can be perceived as shy, coy, bashful, embarrassed. Infant Child Dev. 14, 63–83. 10.1002/icd.379

[B6] Draghi-Lorenz R. Reddy V. Costall A. (2001). Rethinking the development of “nonbasic” emotions: a critical review of existing theories. Dev. Rev. 21, 263–304. 10.1006/drev.2000.0524

[B7] Guillaume P. (1926). L'imitation Chez L'Enfant. Paris: Librairie Felix Alcan.

[B8] Hart S. Carrington H. (2002). Jealousy in 6-month-old infants. Infancy 3, 395–402. 10.1207/S15327078IN0303_6 33451216

[B9] Hart S. L. Carrington H. A. Tronick E. Z. Carroll S. R. (2004). When infants lose exclusive maternal attention: is it jealousy? Infancy 6, 57–78. 10.1207/s15327078in0601_3

[B10] Hoffmann M. L. (1984). Interaction of affect and cognition in empathy, in Emotions, Cognition and Behavior, eds Izard C. E. Kagan J. Zajonc R. B. (Cambridge: Cambridge University Press), 103–131.

[B11] Lewinski P. (2015a). Automated facial coding software outperforms people in recognizing neutral faces as neutral from standardized datasets. Front. Psychol. 6:1386. 10.3389/fpsyg.2015.01386 26441761 PMC4565996

[B12] Lewinski P. (2015b). Don't look blank, happy, or sad: patterns of facial expressions of speakers in banks' YouTube videos predict video's popularity over time. J. Neurosci. Psychol. Econ. 8, 241–249. 10.1037/npe0000046

[B13] Lewinski P. den Uyl T. M. Butler C. (2014a). Automated facial coding: validation of basic emotions and FACS AUs in FaceReader. J. Neurosci. Psychol. Econ. 7, 227–236. 10.1037/npe0000028

[B14] Lewinski P. Fransen M. L. Tan E. S. H. (2014b). Predicting advertising effectiveness by facial expressions in response to amusing persuasive stimuli. J. Neurosci. Psychol. Econ. 7, 1–14. 10.1037/npe0000012

[B15] Lewis M. (1987). Social development in infancy and early childhood, in Handbook of Infant Development, 2nd Edn., ed Osofsky J. (New York, NY: Wiley), 419–493.

[B16] Lewis M. (1993). The emergence of human emotions, in Handbook of Emotions, eds Lewis M. Haviland J. M. (New York, NY: Guildford), 223–235.

[B17] Masciuch S. (1988). Jealousy: the quantitative and qualitative testing of a Kleinian psychoanalytic neo-piagetian paradigm. J. Melanie Klein Soc. 6, 14–34.

[B18] Olszanowski M. Pochwatko G. Kuklinski K. Scibor-Rylski M. Lewinski P. Ohme R. K. (2015). Warsaw set of emotional facial expression pictures: a validation study of facial display photographs. Front. Psychol. 5:1516. 10.3389/fpsyg.2014.01516 25601846 PMC4283518

[B19] Piaget J. (1932). Le Jugement Morale Chez L'Enfant. Paris: Puf.

[B20] Reddy V. (2000). Coyness in early infancy. Dev. Sci. 3, 186–192. 10.1111/1467-7687.00112

[B21] Reissland N. (1990). Parental frameworks of pleasure and pride. Infant Behav. Dev. 13, 249–256.

[B22] Trevarthen C. (1979). Communication and cooperation in early infancy: a description of primary intersubjectivity, in Before Speech: The Beginnings of Human Communication, ed Bullowa M. (London: Cambridge University Press), 321–347.

[B23] Trevarthen C. (1984). Emotions in infancy, in Approaches to Emotions, eds Scherer K. R. Ekman P. (London: Erlbaum), 129–157.

[B24] Trevarthen C. (1992). An infant's motives for speaking and thinking in the culture, in The Dialogical Alternative, ed Wold A. H. (Oslo: Scandinavian University Press), 99–137.

[B25] Walker-Andrews A. S. (1997). Infants' perception of expressive behaviors: differentiation of multimodal information. Psychol. Bull. 121, 437. 9136644 10.1037/0033-2909.121.3.437

